# Increased Level of Long Non-Coding RNA MALAT1 Is a Common Feature of Amoeboid Invasion

**DOI:** 10.3390/cancers12051136

**Published:** 2020-05-01

**Authors:** Ladislav Merta, Aneta Gandalovičová, Vladimír Čermák, Michal Dibus, Tony Gutschner, Sven Diederichs, Daniel Rösel, Jan Brábek

**Affiliations:** 1 Department of Cell Biology, Charles University, Viničná 7, 12843 Prague, Czech Republic; ladislav.merta@natur.cuni.cz (L.M.); aneta.gandalovicova@natur.cuni.cz (A.G.); vladimir.cermak@natur.cuni.cz (V.Č.); michal.dibus@natur.cuni.cz (M.D.); daniel.rosel@natur.cuni.cz (D.R.); 2 Biotechnology and Biomedicine Centre of the Academy of Sciences and Charles University (BIOCEV), Průmyslová 595, 25242 Vestec u Prahy, Czech Republic; 3 Medical Faculty, Martin-Luther-University Halle-Wittenberg, Kurt-Mothes-Str. 3a, 06120 Halle (Saale), Germany; tony.gutschner@uk-halle.de; 4 Department of Thoracic Surgery, Division of Cancer Research, Medical Center—University of Freiburg, Faculty of Medicine, University of Freiburg, German Cancer Consortium (DKTK)—Partner Site Freiburg, Breisacher Str. 115, 79106 Freiburg, Germany; s.diederichs@dkfz-heidelberg.de; 5 Division of RNA Biology & Cancer, German Cancer Research Center (DKFZ), Im Neuenheimer Feld 280, 69120 Heidelberg, Germany

**Keywords:** MALAT1, lncRNA, cancer, amoeboid invasion, mesenchymal invasion, invasion plasticity, melanoma

## Abstract

The ability of cancer cells to adopt various migration modes (the plasticity of cancer cell invasiveness) is a substantive obstacle in the treatment of metastasis, yet still an incompletely understood process. We performed a comparison of publicly available transcriptomic datasets from various cell types undergoing a switch between the mesenchymal and amoeboid migration modes. Strikingly, lncRNA MALAT1 (metastasis-associated lung adenocarcinoma transcript 1) was one of three genes that were found upregulated in all amoeboid cells analyzed. Accordingly, downregulation of MALAT1 in predominantly amoeboid cell lines A375m2 and A2058 resulted in decrease of active RhoA (Ras homolog family member A) and was accompanied by the amoeboid-mesenchymal transition in A375m2 cells. Moreover, MALAT1 downregulation in amoeboid cells led to increased cell proliferation. Our work is the first to address the role of MALAT1 in MAT/AMT (mesenchymal to amoeboid transition/amoeboid to mesenchymal transition) and suggests that increased MALAT1 expression is a common feature of amoeboid cells.

## 1. Introduction

Invasion of cancer cells leading to metastasis is responsible for the vast majority of the 9.6 million cancer-related deaths each year [1,2]. Cancer cells can migrate through the extracellular matrix (ECM) collectively or individually. In the case of individually migrating cells, two modes of invasion have been described—mesenchymal and amoeboid. Mesenchymally migrating cancer cells are characterized by an elongated shape, dependence on integrin-mediated adhesion, and secretion of proteolytic enzymes. In contrast, amoeboid cells are typically more rounded and frequently display intense membrane blebbing. Due to increased intracellular pressure and high actomyosin contractility, these cells “squeeze” themselves through pores in the ECM without the need to form strong focal adhesions or degrade the ECM [3]. The Rho GTPase protein family (especially Rac1, Cdc42, RhoA, and RhoC) are key regulators of cytoskeletal reorganization, which is essential during cell movement. Unsurprisingly, their activity directly affects invasion modes. Upregulation of Rho-ROCK-driven actomyosin contractility promotes the amoeboid invasion mode. On the other hand, Rac1 is the key regulator of mesenchymal invasion, which drives Arp2/3-meditated actin polymerization [4,5,6].

A large hindrance in treatment of cancer metastasis is the ability of invading cells to switch from one mode of invasion to another in response to conditions of the environment or cancer therapy. This phenomenon is called cancer cell invasion plasticity [7] and is one of the reasons why an effective anti-metastatic treatment is still lacking [8]. Experimentally, the mesenchymal–amoeboid transition (MAT) can be induced by reduction of cell–ECM adhesion, loss of ECM proteolysis, enhanced RhoA-ROCK-driven contractility or microtubule destabilization. The reverse process—amoeboid–mesenchymal transition (AMT) is connected with increase of ECM adhesion, proteolytic ECM remodeling, and activation of Rac-induced actin assembly [9]. However, the molecular events underlying the MAT or AMT are insufficiently described. To improve our understanding of the processes, we performed a comparison of previously published datasets from AMT and MAT experiments, which identified metastasis-associated lung adenocarcinoma transcript 1 (MALAT1) to be involved in cancer cell invasion plasticity.

MALAT1, also referred to as NEAT2 (nuclear enriched abundant transcript 2), is a long non-coding RNA (lncRNA) of about 8000 nt in size [10] which shows high primary structure conservation among mammalian species [11]. It was originally discovered as a prognostic marker of metastasis in lung cancer, as its level was increased in metastatic tumors and its high level predicted poor prognosis [12]. Under physiological conditions, the MALAT1 gene is widely expressed and the transcript stays in the cell nucleus [12]. Within the nucleus, MALAT1 is localized in areas called speckles—reservoirs of splicing factors of pre-mRNA [11,13]. Functionally, MALAT1 has been shown to be involved in alternative splicing, regulation of gene expression, and to affect diverse aspects of cell behavior including proliferation and migration [14].

MALAT1 seems dispensable for normal development in mice since no abnormalities were observed after genetic knock-out [15,16]. Interestingly, however, a significant inhibition of both macro and micrometastases formation was observed in mammary carcinoma mouse model with MALAT1 knock-out, making MALAT1 a promising target in cancer therapy [17]. The upregulation of MALAT1 in cancer cells compared to the non-malignant cells was described in different types of primary tumors and also cancer cell lines derived from various tissues [18,19]. However, the role of MALAT1 in cancer is still ambiguous. In most studies, decreased level of MALAT1 is linked with lower proliferation and tumor growth [17,20,21], however, others found no difference after its knock-out [15], and in some works, MALAT1 was identified even as a suppressor of proliferation [22,23]. Moreover, in several studies, MALAT1 down-regulation resulted in a significant decrease of cancer cell migration [20,24,25,26], whereas in a recent study, MALAT1 suppressed the metastatic ability of breast cancer cells [27].

Taken together, MALAT1 has been shown to be an important player in cancer biology. Here, we report MALAT1 levels are differentially associated with the mesenchymal and amoeboid invasion mode, demonstrating its role in cancer cell invasion plasticity.

## 2. Results

### 2.1. Comparison of Transcriptomic Profiles of Amoeboid Cancer Cells and Macrophages Revealed Upregulation of MALAT1

The transitions between amoeboid and mesenchymal invasion modes are still incompletely understood. To identify genes with a potential role in MAT/AMT, we analyzed publicly available datasets from cells undergoing these transitions. For the comparison, we utilized only data sets from cells that have lost epithelial traits or are of mesenchymal origin. In 3D culture, they do not form aggregates and appear as single cells with clearly discernible morphological phenotype (amoeboid or mesenchymal) before and after the transition. Namely, RNA-seq data from the HT1080 fibrosarcoma cell line undergoing MAT in 3D collagen after induction of constitutively active RhoA (icaRhoA) and after treatment with the Src inhibitor dasatinib (DAS) (manuscript in revision). Furthermore, we analyzed microarray data from constitutively amoeboid A375m2 melanoma cells cultured on top of thick, deformable collagen and treated with ROCK kinase inhibitors Y27632 and H1152 (further referred to as ROCKi) to induce AMT [28]. In addition, we included RNA-seq data from M2 macrophages of either amoeboid or mesenchymal morphology due to different stiffness of the surrounding collagen matrix [29].

**Figure 1:**
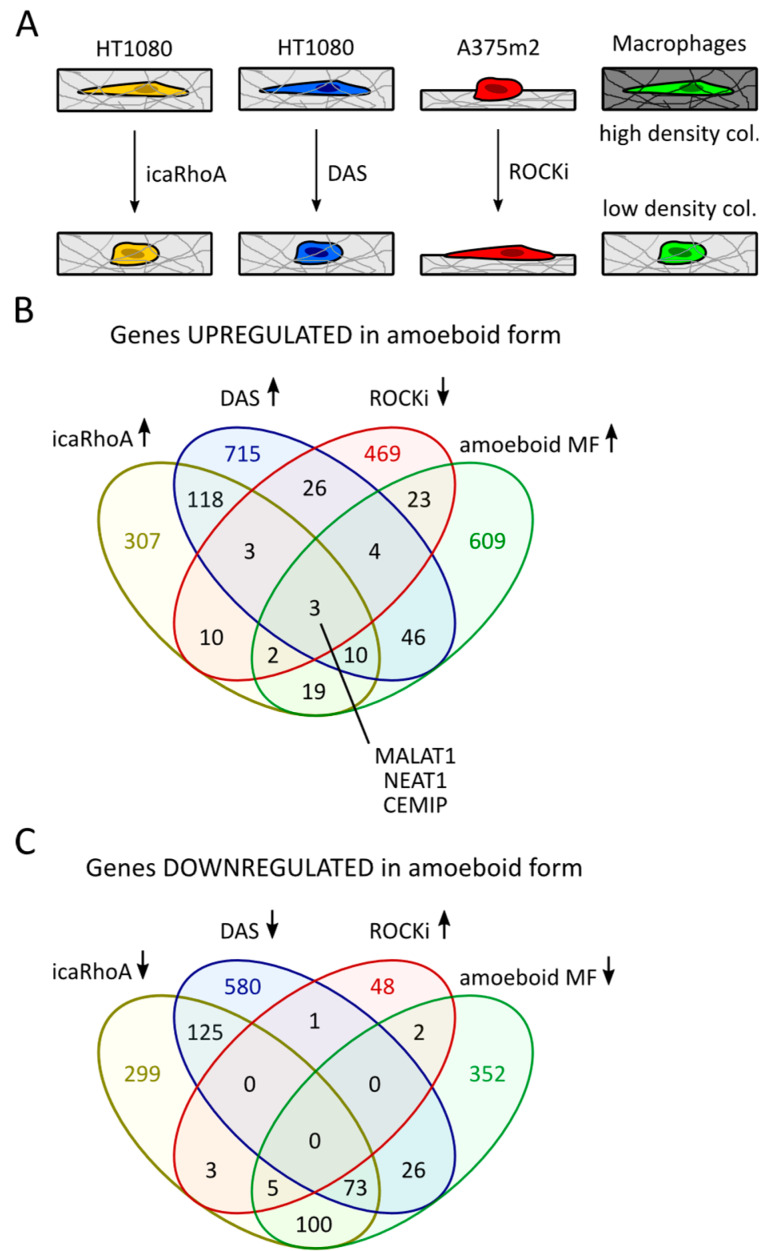
Comparison of published transcriptomic profiles of amoeboid cells in 3D conditions. (**A**) The schematic representations of experimental systems used in the comparison of datasets. (**B**) Venn diagram of gene sets upregulated in the amoeboid HT1080 cells (manuscript in revision) and macrophages [29], and suppressed in A375m2 cells by ROCK inhibitors (ROCKi) [28]. (**C**) Venn diagram of gene sets downregulated in the amoeboid HT1080 cells and macrophages, and upregulated in A375m2 cells by ROCK inhibitors. In all cases, transcripts with adjusted *p*-value ≤ 0.25 and fold change >1.5 in either direction were considered differentially expressed.

To reveal the genes related to amoeboid phenotype, we performed an expression analysis and data intersection of differentially expressed transcripts to identify those genes that were downregulated after AMT (ROCKi treatment), but upregulated after MAT (induced icaRhoA, dasatinib treatment and macrophages in low concentration collagen) (Figure 1A). Strikingly, this analysis revealed only three genes showing consistent upregulation in all amoeboid cells of diverse biological backgrounds—lncRNAs MALAT1 and NEAT1, and CEMIP (Figure 1B). MALAT1 and NEAT1 are both lncRNAs shown to have complementary functions [30], which is why we have focused on these genes in subsequent analysis. CEMIP (cell migration inducing protein) is a protein involved in migration of cancer cells [31] and will be the subject of our future investigation. There was no overlap among genes downregulated in amoeboid cells in the four analyzed datasets (Figure 1C). The overlaps of upregulated/downregulated genes of only three datasets are listed in Supplementary Materials (Figure S1A,B). All data used for the comparison are available as Supplementary File S1.

### 2.2. MALAT1 Level Increases after MAT

**Figure 2:**
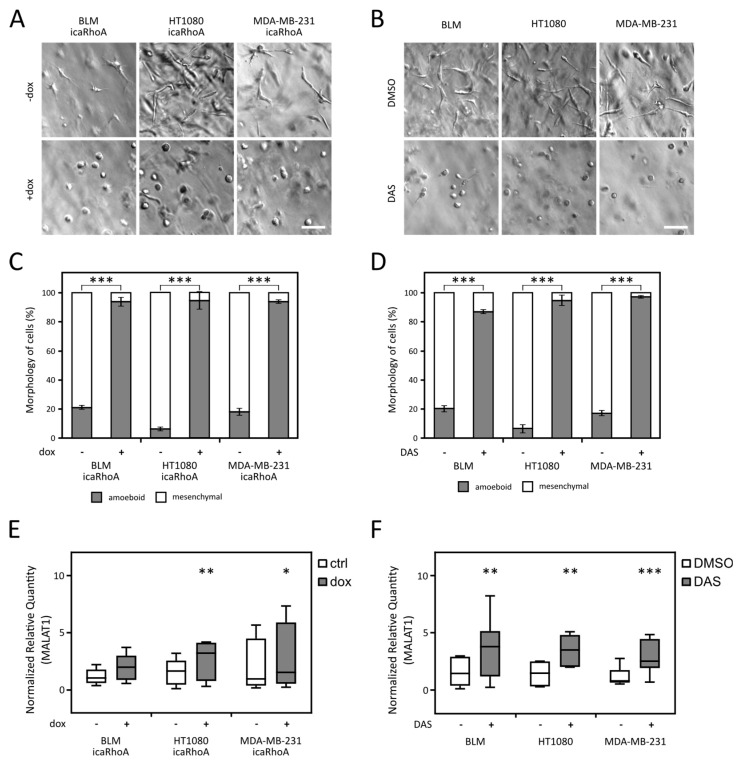
Morphological and RT-qPCR analysis of cell lines undergoing MAT (mesenchymal to amoeboid transition). (**A**,**B**) Representative wide-field images of cells in 3D collagen matrix with or without induction of MAT. (**C**,**D**) Quantification of cell morphology in 3D collagen. Data represent the mean ± SD. (**E**,**F**) RT-qPCR analysis of the MALAT1 (metastasis-associated lung adenocarcinoma transcript 1) gene expression. Median values are marked in the box plots, whiskers represent min to max range. Parts (**A**,**C**,**E**) depict cell lines with inducible constitutively active RhoA used for the MAT induction. (**B**,**D**,**F**) depict cell lines treated with dasatinib (DAS) to induce MAT. *p*-values: *** *p* < 0.001, ** *p* < 0.01, * *p* < 0.05. Scale bar 75 μm in all cases. All data are a representation of at least 3 independent experiments.

LncRNAs MALAT1 and NEAT1 were identified as amoeboid-associated genes by comparison of available datasets. To experimentally verify this finding, we made use of previously established MAT systems in the fibrosarcoma cell line HT1080-doxycycline inducible expression of constitutively active RhoA (icaRhoA) and Src inhibitor dasatinib (DAS) treatment [32,33]. To broaden our results, we analyzed the effect of both MAT-inducing treatments in two additional cell lines with mesenchymal morphology in 3D collagen—BLM (melanoma) and MDA-MB-231 (breast adenocarcinoma). All the cell lines were able to undergo MAT after both treatments (Figure 2A–D).

Next, we analyzed the expression level of both lncRNAs by qPCR after induction of MAT by both treatments in all three cell lines. Interestingly, with the exception of BLM icaRhoA, all other five experimental systems exhibited significantly increased level of MALAT1 lncRNA after MAT (Figure 2E,F). Since the results of NEAT1 gene expression analyses were less consistent (Figure S2A,B), we decided to restrict our further analysis to MALAT1. To rule out the possible expression of a shorter MALAT1 transcript, we also included the analysis of MALAT1 expression using a primer pair targeting a region close to 5’ end of the transcript (Figure S2C,D).

### 2.3. Reduction of MALAT1 Induces AMT in A375m2 Cells and Increases Invasion and Proliferation

**Figure 3:**
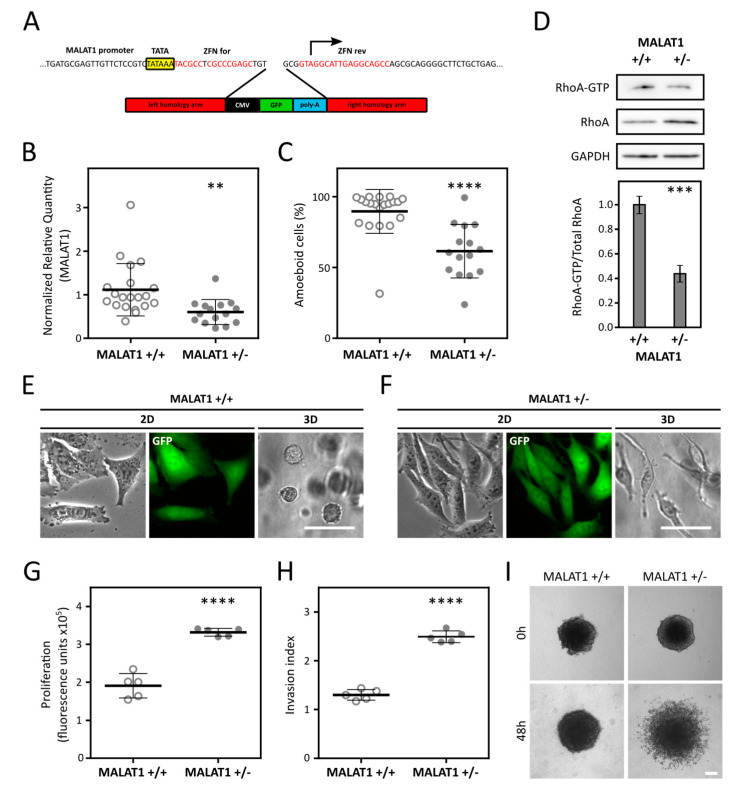
MALAT1 level and morphology of clones derived from the A375m2 cell line. (**A**) Zinc-finger nuclease (ZFN) system for MALAT1 depletion. The zinc-finger nucleases cleave between TATA box (yellow) and the site of transcription start (arrow). The binding motifs for ZFNs are depicted in red. The integration of the cassette into MALAT1 loci is mediated by homologous recombination using left and right homology arm. (**B**) RT-qPCR analysis of the MALAT1 gene expression in A375m2-derived clones. Data represent the mean ± SD. (**C**) Quantification of clones’ morphology in 3D collagen. Data represent the mean ± SD. N MALAT1+/+) = 20 clones; N(MALAT1+/−) = 15 clones. (**D**) Pull-down of active RhoA from 3D samples of pooled clones. Representative immunoblots are in upper part, lower part represents the densitometry quantification. Data represent the mean ± SEM. (**E**) Representative images of a control clone in 2D environment (Petri dish) and in 3D collagen matrix. (**F**) Representative images of a heterozygous clone in 2D environment and in 3D collagen matrix. (**G**) Proliferation of selected clones in 3D collagen. Data represent mean fluorescence of AlamarBlue ± SD. (**H**) Quantification of cell invasion from spheroids. Data represent the mean ± SD. (**I**) Representative images of invasion of control and heterozygous MALAT1 clones from spheroids. *p*-values: **** *p* < 0.0001, *** *p* < 0.001, ** *p* < 0.01. Scale bar 50 μm in parts (**E**,**F**) and 150 μm in part (**I**). Part (**A**) was taken and modified from [34].

As the increased level of MALAT1 expression might be an important feature of amoeboid cells, we further focused on analyzing the possible role of MALAT1 in the induction of the amoeboid phenotype in cancer cells. We wondered if genetic inactivation of MALAT1 can induce AMT in the well-characterized predominantly amoeboid cancer cell line A375m2 [28]. We made use of zinc-finger nucleases (ZFN) and homologous recombination to target the MALAT1 gene by insertional inactivation (Figure 3A). We prepared 35 candidate MALAT1-depleted clones derived from A375m2 cells. Of these, 15 clones showed successful integration of the EGFP expression cassette into MALAT1 locus (heterozygous clones; +/−), while other 20 kept intact MALAT1 alleles and expressed the EGFP gene due to non-specific integration of the cassette outside the MALAT1 locus (wild type clones; +/+). These MALAT wild-type clones were used as controls in subsequent experiments.

We next measured the MALAT1 transcript level in heterozygous and control clones and confirmed that heterozygous clones had significantly lower level of MALAT1 (Figure 3B and Figure S3A). To assess whether reduction of MALAT1 can suppress the amoeboid phenotype of A375m2 cells, we analyzed morphology of the clones in 3D collagen. Indeed, MALAT1+/− clones displayed significantly more elongated (mesenchymal) morphology than the control clones (Figure 3C). The representative morphology of MALAT1+/+ and +/− clones is depicted in Figure 3. To further analyze whether MALAT1+/− clones comply with mesenchymal traits, we have performed an active RhoA pulldown assay using GST-rhotekin bound to glutathione-agarose beads. We selected 5 representative clones of each genotype and pooled them into “+/+” and “+/−” samples. The active RhoA pulldown analysis clearly showed that MALAT1+/− clones had a significantly decreased level of active RhoA (Figure 3D), which is known to accompany AMT. To better characterize the phenotype of the MALAT1 heterozygous clones, we further analyzed the representative 5 clones of each genotype selected for RhoA-GTP pulldown assay. We measured proliferation in 3D collagen using a modified AlamarBlue assay (Invitrogen, Carlsbad, CA, USA) and found that proliferation of MALAT1+/− clones was significantly increased (Figure 3G) compared to control clones. Moreover, we assessed the invasive ability of the clones in 3D collagen using spheroid invasion assay, which showed that the MALAT1+/− clones were significantly more invasive than the MALAT1+/+ clones (Figure 3H,I).

### 2.4. Depletion of MALAT1 in A2058 Cells Decreases RhoA Activation but Is Not Followed by AMT

**Figure 4:**
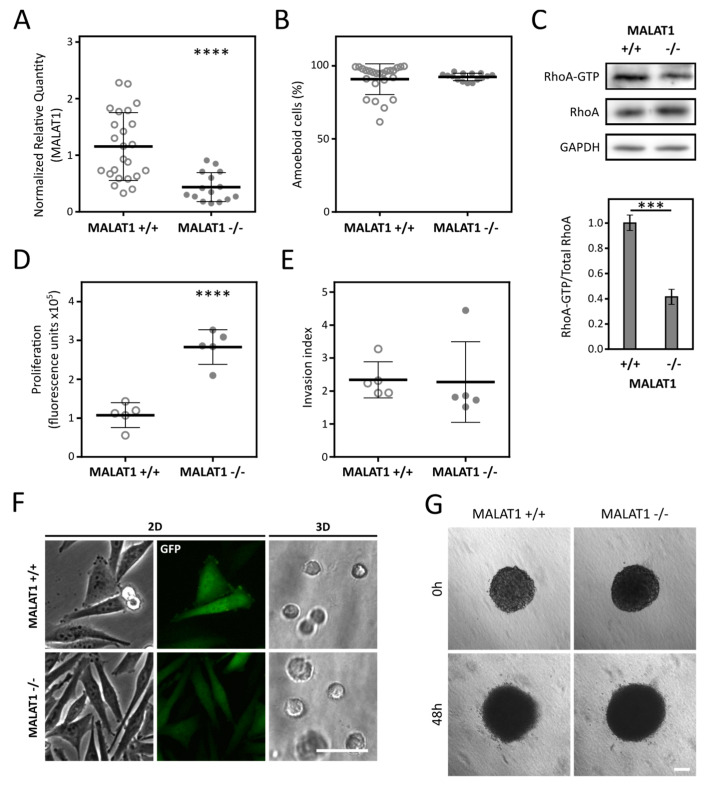
MALAT1 level and morphology of clones derived from the A2058 cell line. (**A**) RT-qPCR analysis of the MALAT1 gene expression in A2058-derived clones. Data represent the mean ± SD. (**B**) Quantification of clones’ morphology in 3D collagen. Data represent the mean ± SD. N(MALAT1+/+) = 24 clones; N(MALAT1−/−) = 15 clones. (**C**) Pull-down of active RhoA from 3D samples of pooled clones. Representative immunoblots are in upper part, lower part represents the densitometry quantification. Data represent the mean ± SEM. (**D**) Proliferation of selected clones in 3D collagen. Data represent mean fluorescence of AlamarBlue ± SD. (**E**) Quantification of cell invasion from spheroids. Data represent the mean ± SD. (**F**) Representative images of MALAT1+/+ and MALAT1−/− clones in 2D environment (Petri dish) and in 3D collagen matrix. (**G**) Representative images of invasion of control and heterozygous MALAT1 clones from spheroids. *p*-values: **** *p* < 0.0001, *** *p* < 0.001, Scale bar 50 μm in part (**F**) and 150 μm in part (**G**).

To broaden our results, we analyzed the effect of MALAT1 inactivation in A2058 melanoma cell line which also exhibits a prevalent amoeboid morphology in 3D collagen [35]. We used the same ZFN-based approach as in A375m2 cells to target the MALAT1 gene in A2058 cells. Interestingly, all the isolated clones that displayed decreased expression of MALAT1 were targeted in both alleles according to the PCR genotyping (MALAT1−/−). Despite the level of MALAT1 being significantly lower in A2058 MALAT−/− clones in comparison with control clones (Figure 4A), all clones maintained their amoeboid phenotype in 3D (Figure 4B). Nevertheless, using the active RhoA pulldown assay in pools of randomly selected 5 “+/+” and 5 “−/−” clones we found that, despite their unchanged morphology, the A2058 MALAT1−/− clones exhibited significantly decreased levels of activated RhoA in 3D when compared to A2058 MALAT1+/+ clones (Figure 4C). Lower levels of active RhoA are supposed to be detected after a switch to mesenchymal phenotype. To further investigate the discrepancy between the decreased levels of Rho activation and the absence of AMT in MALAT−/− A2058 cells, we tested the response of the cells to ROCK inhibitor Y-27632, a well-defined inducer of AMT [3]. We observed no effect after ROCK inhibition (Figure S3B), suggesting that A2058 cells have, at least in part, lost invasion plasticity. Further, the invasiveness of A2058 MALAT1−/− clones was not affected (Figure 4E,G) probably due to their inability to switch to mesenchymal mode of invasion. However, A2058 MALAT1−/− clones were significantly more proliferative than control clones in agreement with the A375m2 results (Figure 4D).

## 3. Discussion

The ability of cancer cells to switch between different modes of invasion due to characteristics of the surrounding environment or in response to therapeutic treatment [36] renders anti-metastatic therapy challenging. Despite the great effort made to explain the invasive behavior of cancer cells, our understanding of its mechanisms is still limited.

In this work we present, to our best knowledge, an unprecedented comparison of parallel mesenchymal and amoeboid transcriptomes was obtained from 3D conditions. Our comparison of publicly available datasets comprised data coming from three diverse cell types and four very different treatments. It is striking that despite the diversity of the datasets, we found an overlap of three genes upregulated in all cells of the amoeboid phenotype—CEMIP and lncRNAs MALAT1 and NEAT1 (Figure 1B). The increased level of MALAT1 after induction of MAT in three distinct cell lines (HT1080 fibrosarcoma cells, MDA-MB-231 breast cancer, and BLM melanoma cell lines) was further experimentally verified by RT-qPCR experiments (Figure 2E,F). It was also previously described in amoeboid macrophages [29].

After confirming MALAT1 upregulation in amoeboid cells, we went further and investigated whether downregulation of MALAT1 could induce AMT in A375m2 melanoma cells with a well-established amoeboid phenotype [37], and also in A2058 cells, another morphologically amoeboid melanoma cell line. We made use of zinc-finger nucleases that were previously used to knock out MALAT1 in A549 lung cancer cells [34] to deplete MALAT1 in A375m2 and A2058 cells. We obtained 15 clones with decreased MALAT1 expression from both cell lines, although, unlike the A2058 clones, we detected only MALAT1+/− clones in case of A375m2 cells, suggesting that MALAT1 may be indispensable for A375m2 cells but dispensable for A2058 cells. This we derive from the fact that A2058 MALAT1−/− cells do not change their phenotype in 3D, and therefore they may withstand the complete loss of MALAT1 in comparison with A375m2 cells which undergo AMT. In agreement with our previous observations of MAT, A375m2 MALAT1+/− clones were significantly more mesenchymal than control ones (Figure 3C) pointing to a regulatory role of MALAT1 in the amoeboid phenotype. We did not observe any morphological change in A2058 MALAT1−/− clones. However, neither did we see any change in response to ROCK inhibition (Figure S3B), commonly used to induce amoeboid-mesenchymal transition [37,38], suggesting A2058 cells, unlike A375m2 cells, do not exhibit invasive plasticity.

Notably, we detected significantly decreased levels of active RhoA in both A375m2 and A2058 clones with decreased MALAT1 expression (Figure 3D and Figure 4C), which is in agreement with previous studies showing MALAT1 level correlates with the levels of RhoA and Rho kinases 1 and 2 [39,40], which are the key regulators of amoeboid phenotype [38]. Nevertheless, the precise role of MALAT1 in amoeboid phenotype regulation is still to be described. Many signaling pathways shown to be associated with amoeboid phenotype induction were correlated with MALAT1. MALAT1 is upregulated in response to hypoxia [41], which induces the amoeboid phenotype [42]. Interestingly, the third gene upregulated in our data analysis—CEMIP, previously described as a migration-promoting gene, was also shown to be upregulated by hypoxia [31]. Furthermore, MALAT1 might be involved in the regulation of innate immune responses as it was found to regulate the expression of interferon-responsive genes and modulate the activity of the NF-κB signaling pathway [43,44]. It is of interest that this signaling pathway also regulates amoeboid invasion [45].

Further characterization of clones derived from both amoeboid cell lines revealed that clones with lower level of MALAT1 were significantly more proliferative compared to control ones (Figure 3G and Figure 4D). Additionally, A375m2 MALAT1+/− clones displayed increased invasion. This may be attributed to their transition to mesenchymal invasion and mechanical properties of the rat tail collagen I which was used in the invasion assays. Rat tail collagen I forms a dense meshwork of thin collagen fibers [46] which can represent a constrictive environment for amoeboid cells but not mesenchymal cells as they actively degrade the ECM to dig their way through it and are not limited by collagen pore sizes.

MALAT1 was also shown in several studies to play a significant role in the process of epithelial to mesenchymal transition (EMT) [47]. EMT is a process during which cells switch into a more pro-invasive mesenchymal phenotype. Further, mesenchymal cells can further switch to the amoeboid phenotype, suggesting MALAT1 as one of the key regulators in the multistep process leading from the epithelial to amoeboid state through EMT and MAT. Unlike our findings, most studies report MALAT1 knockdown to decrease cell proliferation [17,20,21]. We believe that this discrepancy is due to the difference between EMT and MAT processes, and MALAT1 may have different effect on the proliferative ability of mesenchymal and amoeboid cells. The discrepancies may be also due to cell line- or tissue-specific functions of MALAT1 [48,49]. The inconsistent effect of MALAT1 on cell proliferation calls for caution when considering MALAT1 for anti-cancer therapy, as although its downregulation may prevent EMT, it can promote invasion plasticity of individually migrating cells.

Our work is the first to address the role of MALAT1 in MAT/AMT and suggests that increased MALAT1 expression represents a common feature of amoeboid cells. Further studies should be employed to elucidate the molecular mechanisms of the MALAT1 role in amoeboid invasion.

## 4. Materials and Methods

### 4.1. Comparison of Public Transcriptomic Data

Three publicly available datasets were used for analysis: GEO accession GSE23764 [28], ArrayExpress accession E-MTAB-6823 (manuscript in revision), and ArrayExpress accession E-MTAB-6643 [29]. Of the GSE23764 series, the subset of data comprising an AMT experiment was used, i.e., data from control samples and samples treated with ROCK kinase inhibitors Y-27632 and H-1152. Differentially expressed genes were identified using limma R package [50]. Only transcripts affected by both inhibitors were selected for subsequent comparisons. RNA-seq data E-MTAB-6823 and E-MTAB-6643 were analyzed for differential gene expression with the limma-woom algorithm [51]. In all cases, transcripts with adjusted *p*-value ≤ 0.25 were considered differentially expressed. The required minimum fold change was 1.5 in either direction for all the data. The obtained lists of genes were analyzed with Venny 2.1 online tool [52] to find data overlaps.

### 4.2. Cell Lines, Constructs, and Transfection

A375M2 were a kind gift of Prof. Richard Hynes lab, where this cell line was established. A2058 melanoma cells were purchased from ATCC^®^ HTB-43™ (ATCC, Teddington, UK). MDA-MB-231 cells were obtained from ECACC (ECACC, Salisbury, UK) (#92020424). BLM melanoma cell line was kindly provided by L. van Kempen and J.H.J.M. van Krieken, Department of Pathology, Radboud University, Nijmegen Medical Centre, the Netherlands. HT1080 cell line was obtained from dr. Karel Souček, The Institute of Biophysics, Brno, Czech Republic.

All cell lines were cultivated in DMEM (Dulbecco’s Modified Eagle’s Medium medium) supplemented by 10% FBS (fetal bovine serum) and 10 μg/μL ciprofloxacin (Sigma, Piscataway, NJ, USA) in humified atmosphere with 5% CO_2_ at 37 °C. The BLM icaRhoA and MDA-MB-231 icaRhoA cell lines were prepared using pLVX Tet-On Advanced Gene expression system (Takara Bio USA, Inc., Mountain View, CA, USA) as described previously [32] (+manuscript in revision).

### 4.3. MALAT1 Gene Targeting with Zinc Finger Nucleases

A375m2 and A2058 cells were transfected with the ZFN and the homologous recombination construct [34] (see Figure 3A) using PEI transfection reagent (Polysciences, Inc., Warrington, PA, USA); ratio of DNA:PEI was 1:3). Next, EGFP positive cells were sorted into a 96-well plate (1 cell per well) to obtain individual clones. All grown clones were characterized by PCR genotyping and measuring the expression level of MALAT1.

### 4.4. Microscopy of Cells

All images of cells were acquired using Nikon ECLIPSE TE2000-S microscope (Nikon, Tokyo, Japan). Images of cells in collagen were taken using Hoffman modulation contrast (10×/0.25 or 20×/0.40 objectives), and of spheroids using 4×/0.13 objective.

### 4.5. 3D Cell Culture

Cells were brought to suspension, counted, and centrifuged (4 min, 200 rcf, 25 °C). The cell pellets were resuspended, mixed with collagen solution on ice, and plated in wells. After 15 min incubation at 37 °C, the gelled samples were overlaid with cultivation medium containing 1% FBS. The resulting composition of the collagen matrix was 1 mg/mL rat-tail collagen, 1×RPMI medium, 15 mM HEPES, 1% fetal bovine serum, and 50 μg/mL gentamicin. For details about the number of cells and the amount of collagen matrix for respective approaches, see the respective part of the Methods. For induction of inducible constructs (icaRhoA), 250 nM doxycycline (Sigma, Piscataway, NJ, USA) was used. For induction of MAT by dasatinib treatment, 1 μM dasatinib (LC Laboratories, Woburn, MA, USA) was used. The concentration of ROCK inhibitor Y-27632 (Sigma, Piscataway, NJ, USA) was 10 μM.

### 4.6. Morphology of Cells in 3D Collagen

Cells were seeded into collagen matrix (100,000 cells/250 μL of collagen matrix) and cultivated in wells of a 48-well plate. After 48 h, cells were imaged, and morphology of the cells was analyzed using FiJi software. Cells were considered “elongated” when their length/width ratio was greater than 2, otherwise they were considered “rounded”. A minimum of 400 cells was counted per sample. The data were statistically analyzed using Cochran–Mantel–Haenszel test or logistic regression model and Wald test (in case of MALAT1+/− vs +/+ clone groups analysis). Presented data are summarized from at least 3 independent biological experiments—with the exception of morphology analysis of grouped clones (35 clones derived from A375m2 cells), which was carried out in biological monoplicate.

### 4.7. RNA Isolation

Total RNA was extracted from one million cells cultured in 500 μL of 3D collagen gel for 48 h in a 24-well plate (3D) or a semi-confluent 6 cm Petri dish (2D). In the case of 3D isolation, gels from two wells were added to tubes containing 600 μL RNA extraction solution (60% v/v water-saturated phenol, 3.25 M guanidine thiocyanate, 400 mM sodium acetate buffer pH 4.0, 0.4% w/v N-lauroylsarcosine, 160 mM 2-mercaptoethanol), and 100 μL of 6.1 M sodium chloride. Samples were homogenized using Tissue Tearor (BioSpec Products, Bartlesville, OK, USA). For 2D isolation, 600 μL of above-mentioned RNA extraction solution + 400 μL of RNase-free water was added to the dish, incubated for 1 min, and transferred to a 2 mL tube. The following procedure was the same for both cases. Then, 200 μL of chloroform was added and samples were vortexed vigorously for 10 s. After 10-min incubation at room temperature, samples were centrifuged (18,000× *g*, 4 °C, 30 min), upper polar phase was transferred to a fresh tube, the volume was adjusted to 800 μL with RNase-free water, and 600 μL of isopropanol was added to precipitate the RNA. Samples were centrifuged at 18,000× *g*, 4 °C for 10 min, and the resulting pellets were subsequently washed three times with 800 μL of 75% ethanol and air-dried. Finally, RNA was diluted with RNase-free water to final concentration of 0.5 μg/μL.

### 4.8. Reverse Transcription—Quantitative Polymerase Chain Reaction (RT-qPCR)

The RT-qPCR experiments were performed according to MIQE guidelines [53]. RNA reverse transcription was performed as described previously [29] using 3 μg of total RNA in the total volume of 30 μL. The qPCR reaction was performed as described previously [29]. For primer details, see Table S1. Cq values were calculated by setting single threshold value for each target (Bio-Rad CFX Manager 3.1). Cq values were exported and relative expression was calculated using qBase+ 3.1 software (Biogazelle, Zwijnaarde, Belgium) [54] and reference gene indexes determined by geNORM analysis for each cell line and treatment (always 2 reference genes; see Table S2). Amplification efficiencies were determined by the standard curve analysis. The obtained data were statistically analyzed in GraphPad Prism 6 (GraphPad Software, Inc., San Diego, CA, USA) using paired two-tailed *t*-test for MAT qPCR analysis (Figure 2E,F and Figure S2) and unpaired two-tailed *t*-test in the case of MALAT1+/− or −/− vs +/+ clone groups analysis (Figure 3B and Figure 4A).

### 4.9. 3D Invasion Spheroid Assay

Cells were grown as spheroids using a 3D Petri Dish^®^ (Microtissues^®^); #12-81 large spheroids, (Sigma, Piscataway, NJ, USA) according to manufacturer’s protocol for 2 days. The invasion assay was performed in 96-well plates (one spheroid per well, eight technical replicates per sample). Spheroids were embedded into 3D collagen matrix and overlaid with cultivation medium containing 1% FBS. Images of spheroids were taken immediately after embedding into collagen (0 h) and after 48 h (48 h). The area of the spheroids before and after invasion was assessed using FiJi software and the resulting “invasion index” was calculated as the ratio of the area at 48 h/0 h. The data were statistically analyzed in GraphPad Prism 6 using two-way ANOVA. Presented data are summarized from at least 3 independent biological experiments (13–27 individual spheroids per clone).

### 4.10. Proliferation Assay in 3D Collagen

Cell were seeded into collagen matrix (40,000 cells/100 μL of collagen matrix; 4 technical replicates per sample) to a 96-well plate, overlaid by DMEM without phenol red containing 1% FBS (100 μL) and cultivated for 48 h. Collagen without cells served as a blank for the experiment. After 48 h, overlaying medium was replaced by medium containing AlamarBlue reagent (Invitrogen, Carlsbad, CA, USA) in 5:1 ratio and cultivated for another 4 h. Finally, the medium containing AlamarBlue was transferred to new wells and the fluorescence (excitation 550 nm, emission 590 nm) was measured using the Infinite M200 Pro plate fluorimeter (TECAN, Mannedorf, Switzerland). At least three independent experiments were performed and at least 3 technical replicates were analyzed per sample. The data were statistically analyzed in GraphPad Prism 6 using two-way ANOVA.

### 4.11. RhoA-GTP Pulldown Assay

Five clones for respective genotype and cell line chosen for further experiments were pooled (the same number of cells per clone) into “+/+”, “+/−”, and “−/−” samples. One million cells were cultured in a 500 μL 3D collagen gel for 48 h in a 24-well plate. Gels from two wells were transferred to tubes containing 500 μL of 2× Triton-X100 lysis buffer (2% Triton X-100, 100 mM Tris, 300 mM NaCl, pH = 7.1, protease inhibitors) and homogenized using Tissue Tearor (BioSpec Products, Bartlesville, OK, USA) on ice. After 10-min centrifugation (18,000× *g*, 10 °C), 800 μL of the solution was transferred to a fresh tube, protein concentration in the lysate was determined using the DCTM Protein Assay (Bio-Rad Laboratories, Hercules, California, CA, USA) and adjusted to the same value in each series with 1× Triton X-100 lysis buffer. Then, 50 μL of the lysate was transferred to a fresh tube (total lysate control) and the rest was incubated with rhotekin-bound GST-beads at 4 °C for 45 min. Beads were separated by brief centrifugation and washed two times with 1× Triton X-100 lysis buffer. Finally, beads were resuspended in 1× Laemmli sample buffer (0.35 M Tris-HCl, pH = 6.8, 10% SDS, 40% glycerol, 0.012% bromophenol blue) with DTT (50 mM) and incubated at 95 °C for 10 min. Samples were separated on 10% or 12% SDS-polyacrylamide gels and transferred onto nitrocellulose membrane. Non-specific binding was blocked by incubation of the membranes for 60 min at room temperature in Tris-buffered saline (TBS) containing 4% BSA or 5% non-fat dry milk. The membranes were incubated with a primary antibody in 4 °C overnight, washed three times in Tris-buffered saline with Tween-20 (TBST), and incubated for 75 min with HRP-conjugated secondary antibody at room temperature. Membranes were washed with TBST two times, with TBS one time, and developed using Amersham^TM^ Imager 600 (GE Healthcare, Chicago, IL, USA) and SuperSignal^TM^ Femto Maximum Sensitivity Substrate (Thermo Fisher Scientific, Waltham, MA, USA) or Western Bright TMECL (Advansta, San Jose, CA, USA) HRP substrates. For probing the total protein level after a phosphoprotein detection and for loading control (GAPDH) detection, membranes were stripped in stripping buffer (200 mM NaOH) at 42 °C for 10 min. The primary antibodies used were as follows: GAPDH (Thermo Fisher Scientific MA5-15738), RhoA (Cell Signaling Technology #2117). Quantification of band signals was performed using Multi Gauge software (Fujifilm, Tokyo, Japan). Band intensities of specific proteins were normalized to the GAPDH protein signal. The data were statistically analyzed in GraphPad Prism 6 using *t*-test. The uncropped membranes are available in Figure S4.

## 5. Conclusions

In conclusion, we have shown that lncRNA MALAT1 might play an important role in amoeboid invasion. We have identified an increased level of MALAT1 in cells undergoing MAT in 3D collagen matrix. Furthermore, decrease of MALAT1 expression in predominantly amoeboid cells led to lowered RhoA activity (which is a well-established characteristic of AMT), an increase in cell proliferation and in A375m2 cell line also to morphologically manifested AMT with increased invasion. This is to our best knowledge the first work that analyses the role of MALAT1 in MAT/AMT.

## Supplementary Materials

The following are available online at https://www.mdpi.com/2072-6694/12/5/1136/s1, Figure S1: Transcriptomic overlaps of only 3 datasets used for meta-analysis, Figure S2: RT-qPCR analysis of NEAT1 and second pair of primers for MALAT1 in cells undergoing MAT, Figure S3: Complementary RT-qPCR quantification of MALAT1 level in clones derived from A375m2 and A2058 cell lines and quantification of cell morphology of A2058 cells after treatment with Y-27632, Figure S4: Uncropped Western blots used in the study, Table S1: RT-qPCR primers used in the work, Table S2: Reference genes used for respective cell lines in RT-qPCR analysis, File S1: data used for the comparison of transcriptomic profiles.
