# Supplementary material for: Increased Level of Long Non-Coding RNA MALAT1 Is a Common Feature of Amoeboid Invasion
Source: Cancers (Basel). 2020 May 1;12(5):1136. doi: 10.3390/cancers12051136 (PMC7281393; doi:10.3390/cancers12051136)
Supplement: Supplementary file 1 [file cancers-12-01136-s001.zip › cancers-762835-supplementary/cancers-762835-supplementary.pdf]

cancers

MDPI

Article

Increased Level of Long Non-Coding RNA MALAT1 is a Common Feature of Amoeboid Invasion

Ladislav Merta, Aneta Gandalovičová, Vladimír Čermák, Michal Dibus, Tony Gutschner, Sven Diederichs, Daniel Rösel and Jan Brábek

Supplementary Materials

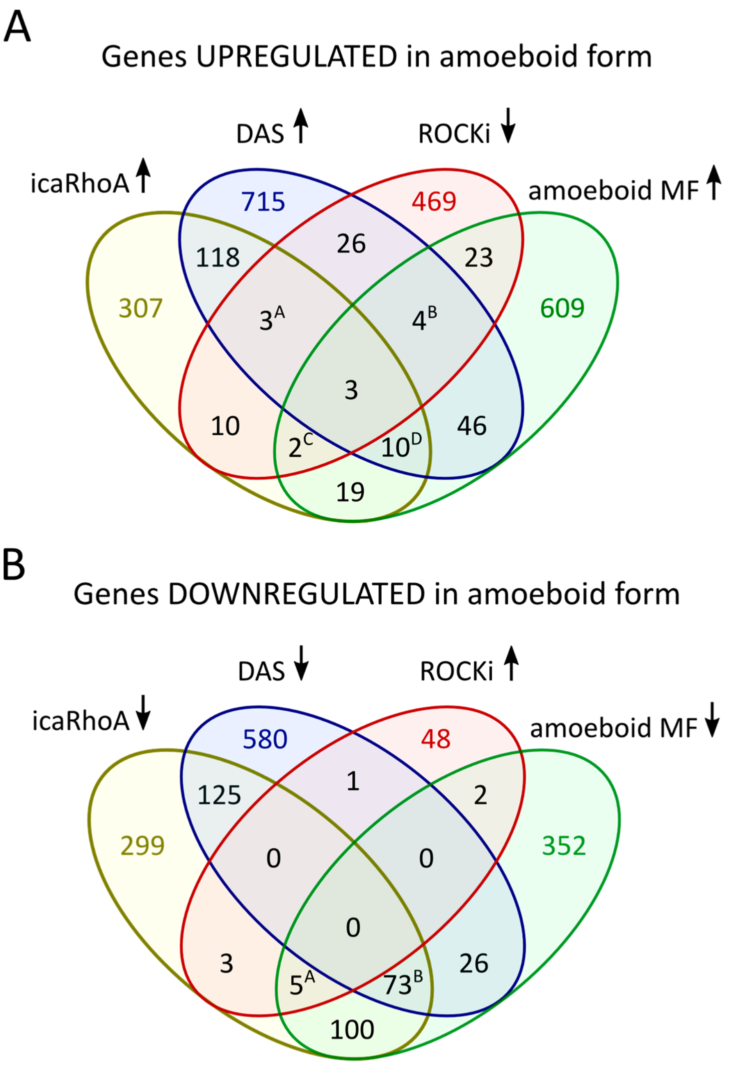

(A)

List A:	List B:	List C:	List D:
CPEB4	ITGA1	FSTL1	KYNU
IL24	MMP1	SLAMF7	LAMA1
SLC30A1	NR4A2		MCTP2
	NR4A3		SCG5
			SERPINB2
			SLC12A8
			STC2
			SULT1C2
			TMTC1
			WDR66

(B)

List A:	List B:			
BIRC5	ANLN	CEP55	MAD2L1	SGO1
CDCA3	APOBEC3B	CIT	MELK	SKA1
SPC24	BUB1	CKAP2L	MND1	SKA3
TK1	BUB1B	CKS1B	NCAPG	STIL
TROAP	C4orf46	DEPDC1	NDC80	STMN1
	CCNA2	DEPDC1B	NEIL3	TCF19
	CCNB1	DIAPH3	NEK2	TMEM106C
	CCNB2	DLGAP5	NUF2	TMEM97
	CDC25C	DTL	NUSAP1	TPX2
	CDC45	FBXO5	PLK4	TRIP13
	CDC6	GINS2	POLE2	TTK
	CDCA7	HELLS	PRC1	TUBA1A
	CDK1	HJURP	PRIM1	UBE2C
	CDKN3	HMMR	PRR11	UBE2T
	CENPH	IQGAP3	PTTG1	WDR76
	CENPI	KIF15	RAD51	ZNF367
	CENPK	KIF2C	RAD51AP1	
	CENPQ	KNL1	RMI2	
	CENPU	LMNB1	RPA3	

Figure 1. Transcriptomic overlaps of only 3 datasets used for meta-analysis. (A) Venn diagram of gene sets upregulated in the amoeboid HT1080 cells and macrophages, and suppressed in A375m2 cells by ROCK inhibitors (ROCKi). (B) Venn diagram of gene sets downregulated in the amoeboid HT1080 cells and macrophages, and upregulated in A375m2 cells by ROCK inhibitors. Lists of the genes are provided for overlaps of 3 gene sets.

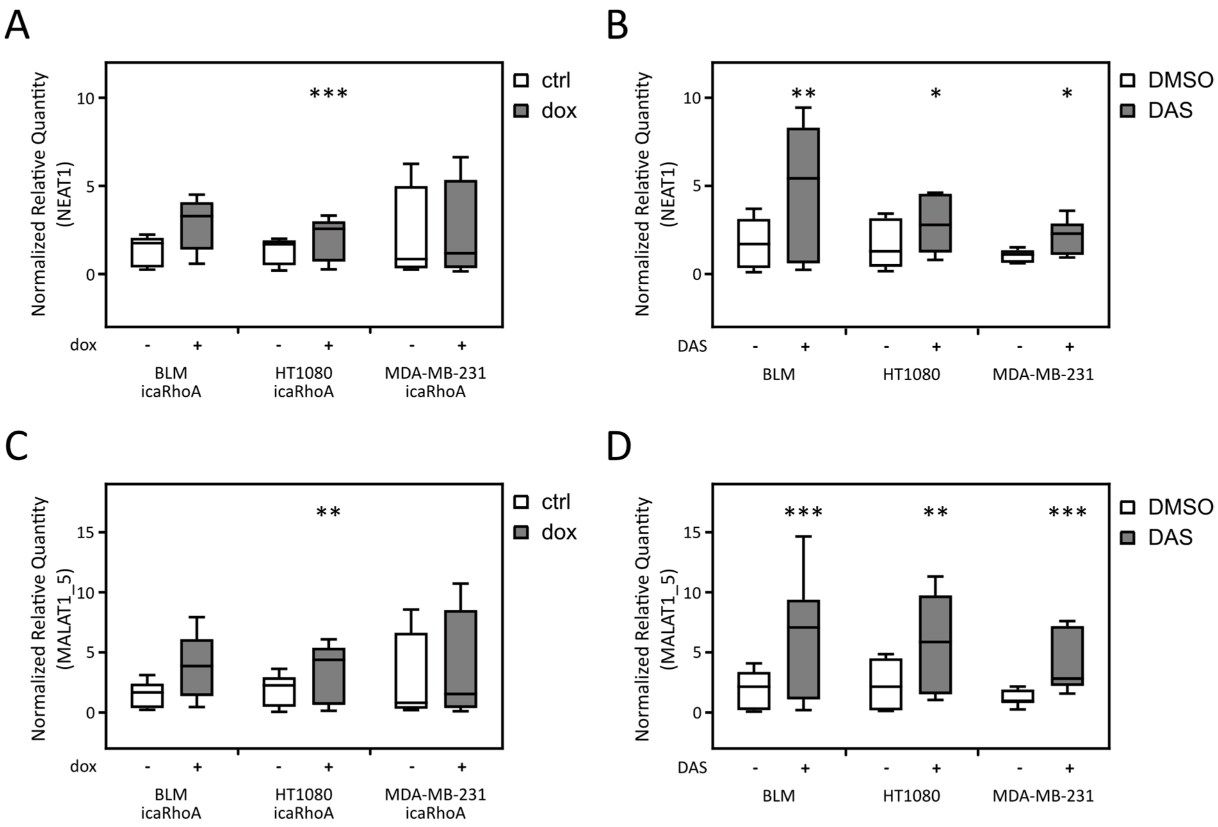

Figure 2. RT-qPCR analysis of NEAT1 and second pair of primers for MALAT1 in cells undergoing MAT. (A-B) Analysis of the NEAT1 gene expression in cells undergoing MAT by induction of constitutively active RhoA (A) or by dasatinib treatment (B). (C-D) Analysis of the MALAT1 gene expression (using primer pair pairing with 5′ end of the transcript) in cells undergoing MAT by induction of constitutively active RhoA (C) or by dasatinib treatment (D). Median values are marked in the box plots, whiskers represent min to max value. P-values: *** p<0.001, ** p<0.01, * p<0.05. All data are a representation of at least 3 independent experiments.

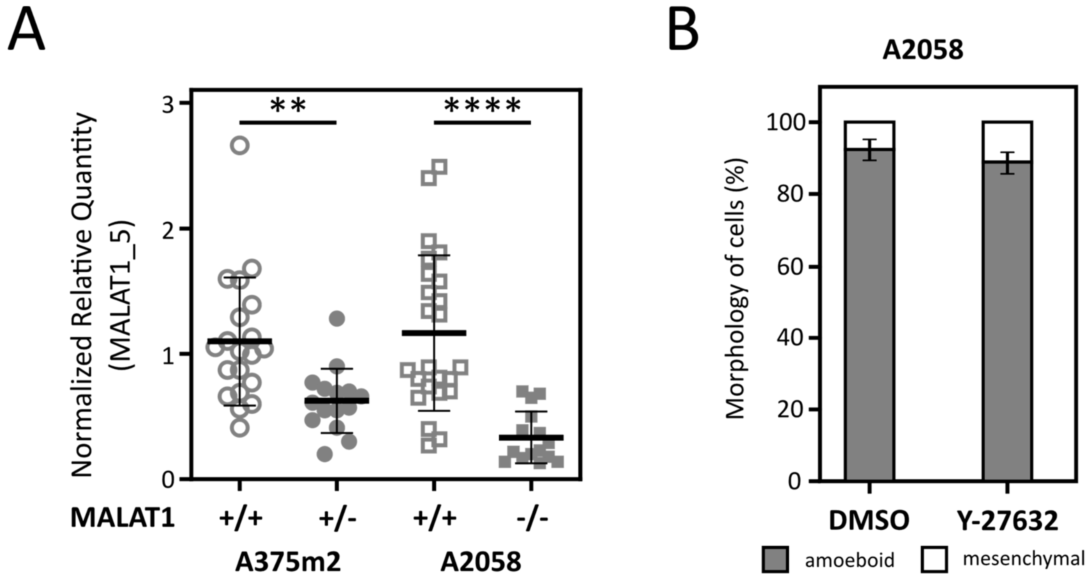

Figure 3. Complementary RT-qPCR quantification of MALAT1 level in clones derived from A375m2 and A2058 cell lines and quantification of cell morphology of A2058 cells after treatment with Y-27632. (A) RT-qPCR analysis of MALAT1 level in clones derived from the A375m2 and A2058 cell lines using second pair of primers pairing with the 5′ end of MALAT1 transcript. N(A375m2 MALAT1 +/+) = 20 clones; N(A375m2 MALAT1 +/-) = 15 clones; N(A2058 MALAT1 +/+) = 24 clones; N(A2058 MALAT1 -/-) = 15 clones. Data represent mean ± SD. (B) morphology of A2058 cells after treatment with ROCK inhibitor Y-27632. Data represent mean ± SD. P-values: **** p<0.0001, ** p<0.01. All data are a representation of at least 3 independent experiments.

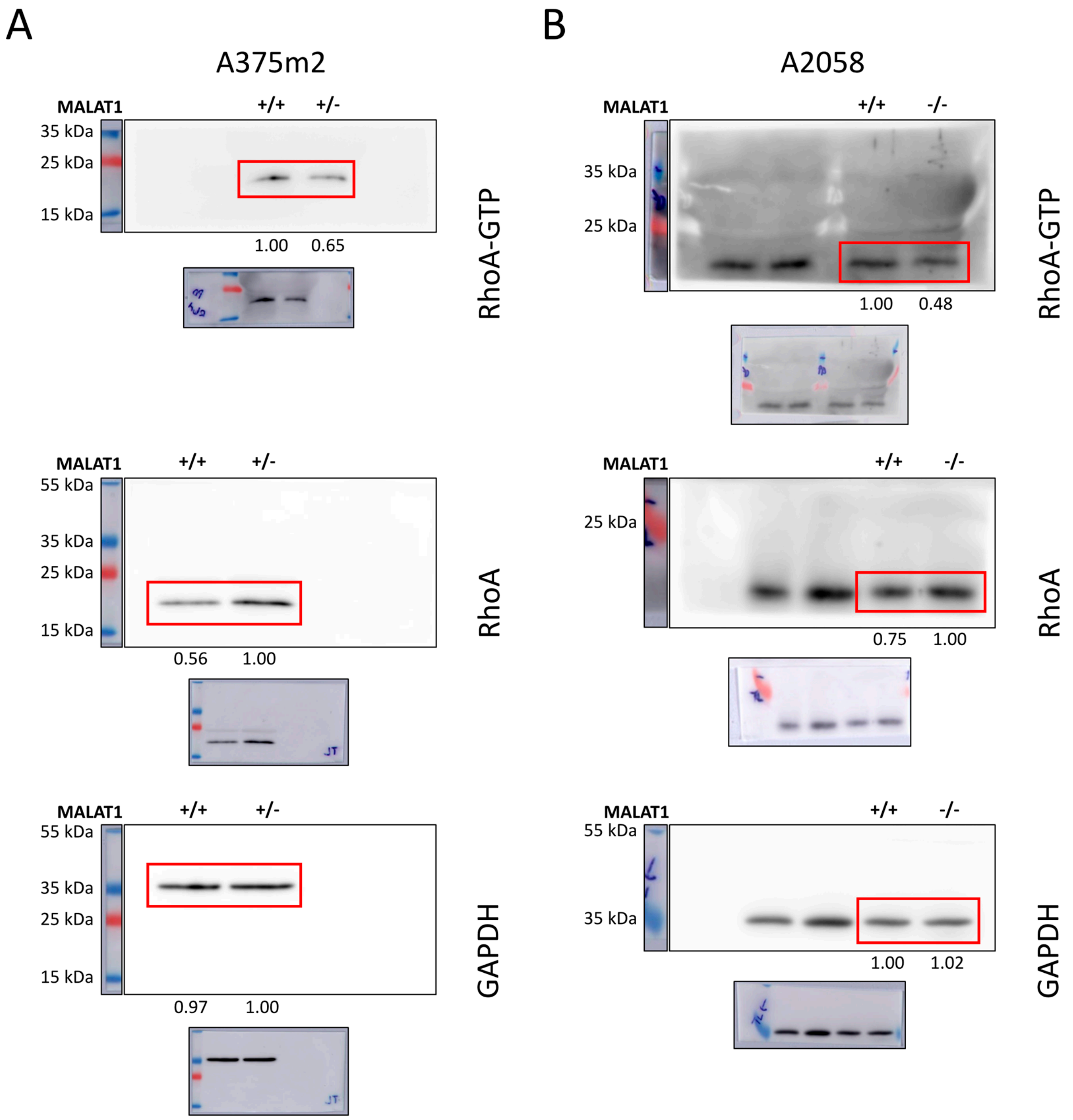

Figure 4. Uncropped Western blots used in the study. (**A**) Uncropped blots used in Figure 3D. (**B**) Uncropped blots used in Figure 4C. Red rectangle marks the area presented in the main text. The numbers below the bands represent the intensity ratio. Because the machine used to visualize the blots uses different channels of photo acquisition, each part consists of the HRP signal (the channel from which the cropped images were made) + added marker (upper part) and the whole membrane HRP signal and transmitted light composite channel (lower part).

Table 1. RT-qPCR primers used in the work.

TRANSCRIPT	ACC. NUMBER	PRIMER	SEQUENCE	nt	%GC	T_m (°C)	PRODUCT (bp)	Eff
**EIF4H**	**NM_022170**	**EIF4Ha**	CCTTCTGGCTACGGGAACCAT	21	57.1	63.3	95	1.977
		**EIF4Hs**	CTTCGACACCTACGACGATCG	21	57.1	63.3		
**GAPDH**	**NM_002046**	**GAPDHa**	GCATGGACTGTGGTCATGAG	20	55.0	60.5	87	2.012
		**GAPDHs**	CTGCACCACCAACTGCTTAG	20	55.0	60.5		
**HNRNPL**	**NM_001533**	**HNRNPLa**	GCGCTCACTTTTGCCTGAGAA	21	52.4	61.3	96	1.993
		**HNRNPLs**	CTGGAGGTGACCGAGGAGAA	20	60.0	62.5		
**MALAT1**	**NR_002819**	**MALAT1_3a**	GGTCTGTGCTAGATCAAAAGG	21	47.6	59.4	71	2.010
		**MALAT1_3s**	AAAGCAAGGTCTCCCCACAAG	21	52.4	61.3		
**MALAT1_5**	**NR_002819**	**MALAT1_5a**	GTTTCATCCTACCACTCCCAAT	22	45.5	60.3	85	1.992
		**MALAT1_5s**	GAATTGCGTCATTTAAAGCCTA	22	36.4	56.5		
**NEAT1**	**NR_131012**	**5NEAT1f**	GTGGTAGGAAATGCAGGTTGA	21	47.6	59.4	122	1.904
		**5NEAT1r**	CCAAGCAACAACTTAACCAACA	22	40.9	58.4		
**PPIA**	**NM_021130**	**PPIAf**	GCCGAGGAAAACCGTGTACTA	21	52.4	61.3	106	1.941
		**PPIAr**	CTGCAAACAGCTCAAAGGAGAC	22	50.0	62.1		
**PSMA1**	**NM_148976**	**PSMA1a**	AGACCAACTGTGGCTGAACCT	21	52.4	61.3	95	1.945
		**PSMA1s**	GACAATGATGTCACTGTTTGGAG	23	43.5	61.1		

Nt; Number of nucleotides, Tm; Melting temperature, Eff; Amplification efficiency determined by standard curve analysis.

Table 2. Reference genes used for respective cell lines in RT-qPCR analysis.

Cell line/clones derived from cell line	Reference genes
BLM (DMSO/DAS)	EIF4H, GAPDH
BLM icaRhoA (-dox/+dox)	EIF4H, HNRNPL
HT1080 (DMSO/DAS)	EIF4H, GAPDH
HT1080 icaRhoA (-dox/+dox)	EIF4H, GAPDH
MDA-MB-231 (DMSO/DAS)	HNRNPL, PSMA1
MDA-MB-231 (-dox/+dox)	EIF4H, HNRNPL
A375m2 clones	EIF4H, PPIA
A2058 clones	PPIA, PSMA
